# Advances of berberine against metabolic syndrome-associated kidney disease: Regarding effect and mechanism

**DOI:** 10.3389/fphar.2023.1112088

**Published:** 2023-02-06

**Authors:** Ya-Fei Liu, Huan-Huan Wang, Yin-Hong Geng, Liang Han, Sheng-Hao Tu, Hui Wang

**Affiliations:** ^1^ Department of Nephrology, The First Affiliated Hospital of Zhengzhou University, Zhengzhou, Henan, China; ^2^ Institute of Integrated Traditional Chinese and Western Medicine, Tongji Hospital, Tongji Medical College, Huazhong University of Science and Technology, Wuhan, Hubei, China; ^3^ Nephrology Department of Integrated Traditional Chinese and Western Medicine, The First Affiliated Hospital of Zhengzhou University, Zhengzhou, Henan, China

**Keywords:** berberine, metabolic syndrome, obesity, diabetic nephropathy, insulin resistance, hyperlipidemia, kidney disease

## Abstract

The prevalence of metabolic syndrome (MetS) is drastically growing worldwide, resulting in MetS-associated kidney disease. According to traditional theories, preventing blood pressure, lipid, glycose, and obesity and improving insulin resistance (IR), a couple of medications are required for MetS. It not only lowers patients’ compliance but also elevates adverse reactions. Accordingly, we attempted to seek answers from complementary and alternative medicine. Ultimately, berberine (BBR) was chosen due to its efficacy and safety on MetS through multi-pathways and multi-targets. The effects and mechanisms of BBR on obesity, IR, diabetic nephropathy, hypertension, hyperlipidemia, and hyperuricemia were elaborated. In addition, the overall properties of BBR and interventions for various kidney diseases were also collected. However, more clinical trials are expected to further identify the beneficial effects of BBR.

## 1 Introduction

With socioeconomic advances and altered lifestyles, metabolic syndrome (MetS) has gained increasing attention and was first defined by a Swedish physician in the 1920s (Studien, 1923). Although the diagnostic criteria of MetS proposed by various agencies, such as the National Cholesterol Education Program’s Adult Treatment Panel III (NCEP-ATP III), the International Diabetes Federation (IDF), the World Health Organization, and the Chinese Diabetes Society (CDS), differ, and abdominal obesity and insulin resistance (IR) are the common denominators. MetS is characterized by IR, obesity, hyperglycemia, hyperlipidemia, and hypertension. It is initiated by abdominal obesity and centered on IR. In other words, visceral obesity leads to IR, which leads to abnormal components of MetS.

MetS is approximately three times more common than diabetes, and the estimated global prevalence is approximately one-quarter of the world population (Saklayen, 2018). Currently, a growing number of studies have reported that obesity (Xu et al., 2021), hypertension (Sun et al., 2020), diabetes, and hyperlipidemia (Qian et al., 2021) can induce a wide range of kidney diseases. Therefore, MetS is prone to cause kidney injury. To our knowledge, MetS-associated kidney injury was first recorded by a Chinese physician in 2002 (Yun-Kai et al., 2002). Interventions for MetS-associated kidney disease include weight loss, lowering hypertension, glucose control, lifestyle improvement, lipid adjustment, Chinese herbal medicines, probiotics, and stem cell therapy (Lin et al., 2022). These therapies are aimed at different components of MetS, resulting in the prescription of many medications for patients with MetS. Hence, exploring novel therapeutic strategies is necessary. Inspired by the holistic concept of Traditional Chinese Medicine, natural-derived medicine was further investigated.

**FIGURE 1:**
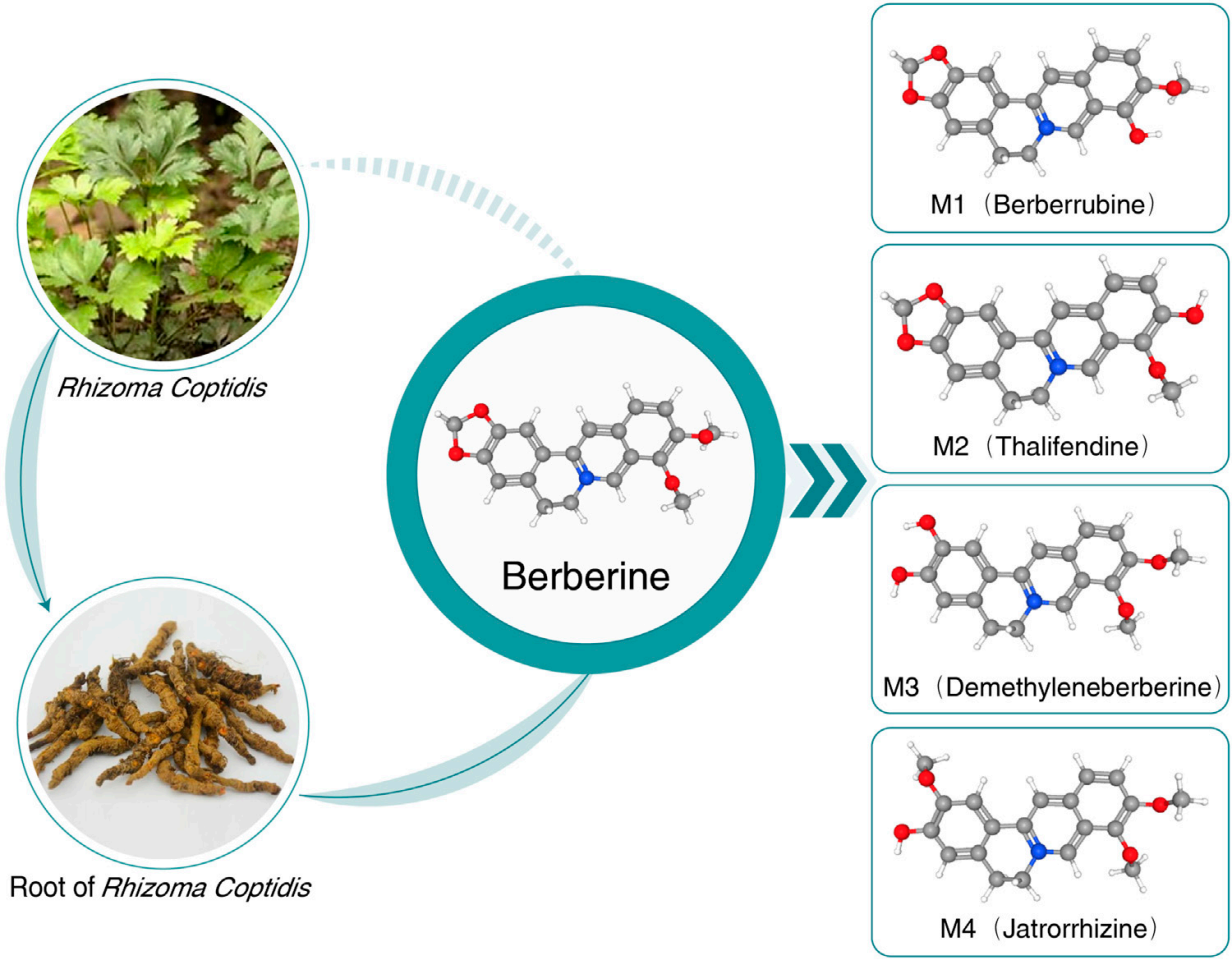
Chemical structure and metabolites of BBR. BBR, berberine; MetS, metabolic syndrome.

After retrieving numerous studies, berberine (BBR) is highlighted due to its wide spectrum of pharmacological activities and intervention mechanisms. The earliest information on the medical use of *Rhizoma coptidis* containing BBR dates back to A.D. 200 (Feng et al., 2019). BBR is a plant isoquinoline alkaloid widely applied in Chinese and Ayurvedic medicine and is extracted from various plants of the Berberidaceae, Ranunculaceae, and Papaveraceae families, such as *Berberis aristata* (5% in roots and 4.2% in stem bark), *Berberis petiolaris* (0.43%), *Berberis vulgaris* (1.24%), *Berberis aquifolium*, *Berberis thunbergii*, *Berberis asiatica*, *Coptis teeta* (rhizome 8%–9%), *Hydrastis canadensis*, *Coptis chinensis*, *Phellodendron amurense*, and *Caulis mahoniae* (Singh and Mahajan, 2013). Recently, most oral BBR has been found in its synthetic form of chloride or sulfate. BBR is a yellow powder that is bitter and slightly soluble in water and ethanol (Kumar et al., 2015). The chemical structure and metabolites of BBR are shown in Figure 1.

**FIGURE 2:**
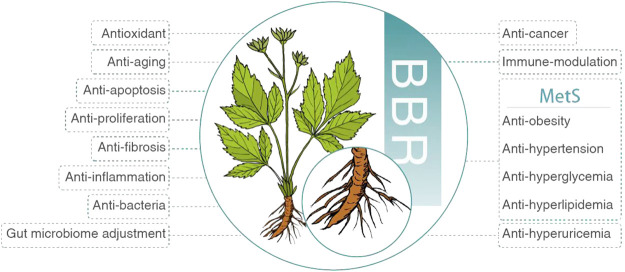
Pharmacological properties of BBR. BBR, berberine; MetS, metabolic syndrome.

Initially, BBR was applied to treat diarrhea and gastrointestinal infection (Yu et al., 2020). Afterward, numerous studies demonstrated its weight loss (Park et al., 2020), antihypertensive (Ma et al., 2017), glucose-lowering (Di et al., 2021), lipid-lowering (Pirillo and Catapano, 2015), uric acid-lowering (Li X. et al., 2021), anti-inflammatory (Oshima et al., 2018; Lu et al., 2022), antifibrotic (Ahmedy et al., 2022), antiproliferative (Bonon et al., 2013), antiapoptotic (Hu et al., 2012), antiaging (Xu Z. et al., 2017), antioxidant (Shou et al., 2022), antibacterial (Liao et al., 2020), anticancer (Liu et al., 2022), immunomodulatory (Huang et al., 2021), gut microbiome adjustment (Wang H. et al., 2022), neuroprotective (Domitrović et al., 2013), cardioprotective (Cai et al., 2021), and neuroprotective (Shou et al., 2022) effects (Figure 2). According to these pharmacological properties, BBR is promising in treating various diseases, such as MetS (Och et al., 2022), hyperuricemia (Li X. et al., 2021), acute kidney injury (Shen et al., 2020), rheumatoid arthritis (Huang et al., 2021), cardiovascular disease (Feng et al., 2019), gastric cancer (Liu et al., 2022), neuropsychiatric disorder (Hao et al., 2022), and polycystic ovary syndrome (Zhang et al., 2021).

**FIGURE 3:**
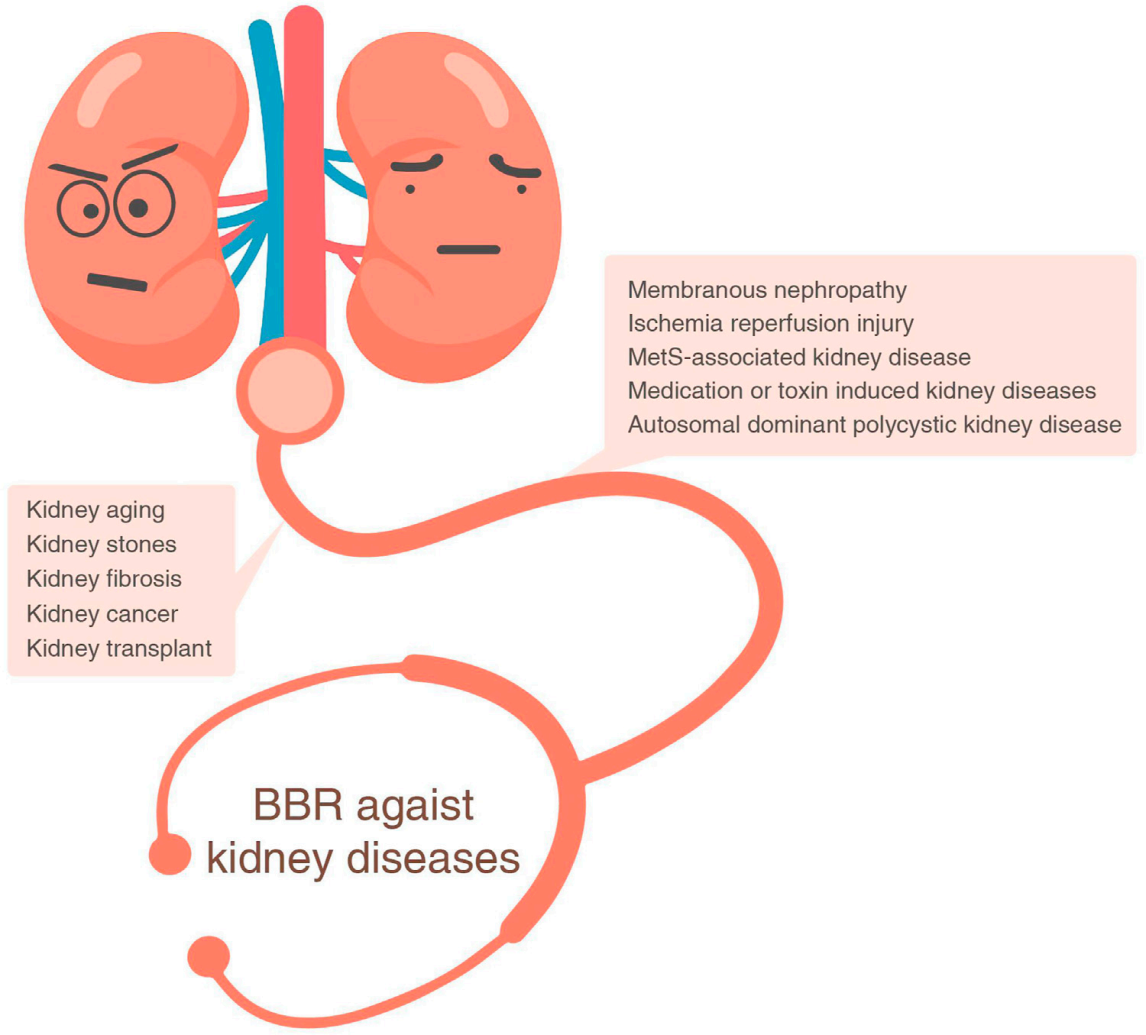
BBR against kidney diseases. BBR, berberine.

Regarding nephroprotective effects, a wide range of kidney diseases could be treated by BBR, such as MetS-associated, membranous nephropathy (Sha et al., 2018), kidney transplant (Wu et al., 2005), ischemia-reperfusion injury (Zheng et al., 2018), kidney fibrosis (Shao et al., 2021), medication- or toxin-induced injury (Othman et al., 2014; Ibrahim Fouad and Ahmed, 2021), autosomal dominant polycystic kidney disease (Bonon et al., 2013), kidney stones (Bashir and Gilani, 2011), kidney aging (El-Horany et al., 2020), and Wilms’ tumor (Liu and Liu, 2013) (Figure 3).

**FIGURE 4:**
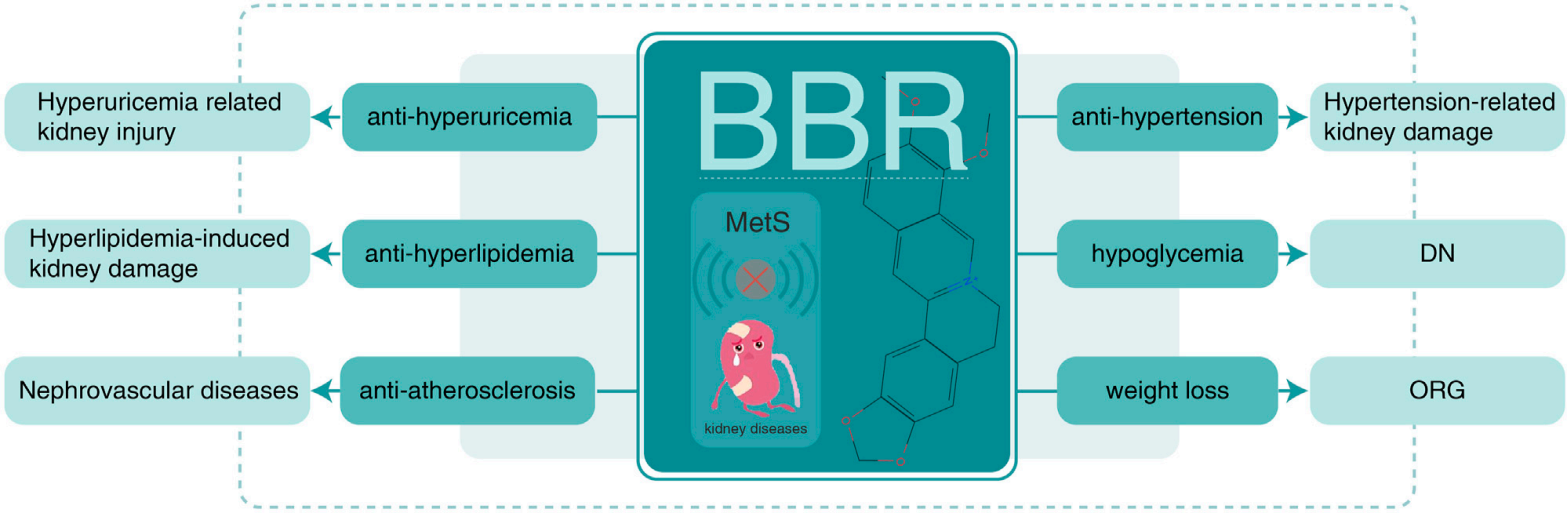
BBR on MetS-associated kidney disease. BBR, berberine; MetS, metabolic syndrome; ORG, obesity-related glomerulopathy; DN, diabetic nephropathy.

In contrast to chemical drugs, which act on special targets and treat unique diseases, herb-derived BBR is multipotent with multiple targets, consistent with the holistic concept of Traditional Chinese Medicine. Consequently, the potential therapeutic action and mechanism of BBR in MetS-associated kidney injury requires summarizing knowledge and research trends (Figure 4).

### 1.1 BBR and obesity

Obesity prevalence is progressively growing as living conditions rise and physical labor decreases. Obesity is most likely caused by a complex mix of changes in the dietary environment; physical activity; and socioeconomic, environmental, and genetic variables. Since 1980, the global prevalence of being overweight and obese has more than doubled, with over a third of the world’s population categorized as overweight or obese. Obesity rates have risen across all ages and sexes, regardless of geographical location, race, or financial level (Chooi et al., 2019).

Obesity-related glomerulopathy (ORG) is becoming increasingly common with the global obesity epidemic. Glomerular hypertrophy and focal segmental glomerulosclerosis (FSGS), particularly the perihilar form, are pathologic characteristics of ORG, and the degree of foot process effacement in ORG is generally less than that in primary FSGS. Obesity-induced increases in glomerular filtration rate, renal plasma flow, filtration fraction, and tubular sodium reabsorption cause the glomerulus to expand. Although most patients have stable or slowly progressing proteinuria, up to one-third suffer progressive renal failure and end-stage renal disease (D'Agati et al., 2016). Insights from Mendelian randomization and human kidney transcriptomics suggest that a putatively causal association of obesity with renal health is primarily independent of blood pressure and type 2 diabetes and reveal the signatures of obesity on the transcriptome of the human kidney (Xu et al., 2021).

BBR, as a botanical compound, is widely employed in ameliorating obesity, although no available research has been mentioned regarding its usage in ORG. To date, many previous studies have demonstrated that BBR effectively inhibits the development of obesity *via* modulation of the gut microbiota, intestinal permeability, gene regulation, and hepatic gluconeogenesis (Ilyas et al., 2020). The detailed potential mechanisms of BBR against obesity are summarized in Table 1 and are as follows.

**TABLE 1:** BBR and obesity.

Author ID	Year	Country	Model	Targeted molecular	Mechanism
Siyu Sun	2022	China	HFD-induced mice, tuft cells	GLP-1, IL-25↑	TAS2R-IL-25
JiWon Noh	2022	Korea	HFD-induced mice	TNF-a, CCL2, CCL3, CCL5, and CXCR4↓	Modulating ATM recruitment and polarization *via* chemotaxis inhibition
HyunJung Park	2020	Republic of Korea	HFD-induced mice	Leptin↓	Controlling the central obesity-related pathway
JianHui Xu	2017	China	HFD-induced rats	IL1beta, TNF-a, PAI-1, NADPHox, STAMP-2, MCP-1, F4/80↓	Modulating gut microbiota and restoring gut barrier
				Claudin-1, ZO-1, GLP-1, GLP-2, GIP, PP, PYY, and colonic proglucagon↑	Improving gut peptide levels
TingGuo	2016	China and United States	HFD-induced mice NAFLD	JNK1 phosphorylation, M2 macrophage, IL-1-beta, TNF-a↓	Anti-inflammation, independent of AMPK
				Adiponectin, M1 macrophage↑	
XuZhang	2012	China	HFD-induced rats	NA	Reducing the exogenous antigen and elevating SCFA levels
WeiDong Xie	2011	China	HFD-induced mice	Fiaf, AMPK, PGC1α, UCP2, CPT1α,and Hadhb↑	Regulating gut microbes
YueShan Hu	2010	United States	HFD-induced mice	PPAR-gamma↓, GATA-3↑	Inhibiting adipogenesis

First, the modulation of gut microbiota: the imbalance of intestinal flora is an important cause of obesity. BBR could reduce the exogenous antigen load in the host and elevate short-chain fatty acid levels in the intestine (Zhang et al., 2012), modulate gut microbiota, restore the gut barrier, and improve gut peptide levels (Xu J. H. et al., 2017).

Second, anti-inflammatory effects: long-term chronic inflammation exists in obesity. BBR treatment notably decreases the phosphorylation state of JNK1 in both hepatoma H4IIE cells and mouse primary hepatocytes, suggesting that improving diet-induced obesity is largely attributable to BBR’s suppression of inflammation (Guo et al., 2016). Furthermore, BBR significantly increases the CD206 + M2 adipose tissue macrophage (ATM) population while significantly reducing tumor necrosis factor-α (TNF-α), C-C motif chemokine ligand 2 (CCL2), CCL4, CCL5, and C-X-C motif chemokine receptor 4 (CXCR4) (Noh et al., 2022).

Third, mitochondrial energy metabolism is regulated. BBR significantly increases fasting-induced adipose factor (Fiaf, a key protein negatively regulated by intestinal microbes) expression in intestinal and visceral adipose tissues. BBR considerably increases AMP-activated protein kinase (AMPK) mRNA, peroxisome proliferator-activated receptor gamma (PPARG) coactivator 1-alpha (PGC1α), uncoupling protein 2 (UCP2), carnitine palmitoyl transferase 1-alpha (CPT1α), and Hadhb related mitochondrial energy metabolism (Xie et al., 2011).

Fourth, the central obesity-related pathway is regulated. By regulating the central obesity-related pathway, microinjections of BBR in the hypothalamus area of rats lower food intake and glucose rise and prevent obesity (Park et al., 2020).

Finally, there are other mechanisms. Because BBR lowers mouse weight gain and food intake with the downregulation of PPAR and upregulation of GATA binding protein 3 (GATA-3), transcription factors function in high-fat diet (HFD)-induced mice (Hu and Davies, 2010). Additionally, through the bitter-taste receptor (TAS2R) signaling pathway, BBR increases the production of glucagon-like peptide-1 (GLP-1) *in vivo* and *ex vivo*, encourages tuft cell proliferation, and secretes IL-25 in obesity (Sun et al., 2022).

### 1.2 BBR and IR

IR is generally secondary to obesity or diabetes. Although it does not directly affect the kidney, its complications, such as MetS, obesity, hypertension, and diabetes, could result in a series of kidney impairments. In the case of these potential kidney injuries, it is imperative to intervene in IR from the origin. BBR is suggested to play a prominent role in attenuating IR *via* different signaling pathways in the cell, animal, and clinical trials involving multiple molecules, proteins, and multiple tissues. The detailed potential mechanisms of BBR against IR are summarized in Table 2 and are as follows.

**TABLE 2:** BBR and IR.

Article ID	Year	Country	Model	Targeted molecular	Pathway
Wenguang Chang	2013	China	H9c2 cardiomyocyte	AMPK↑	Increased AMPK activity
Yanfeng Chen	2009	China	L6 myotubes	PPAR-gamma, FAT/CD36↓	Reducing PPAR-gamma and FAT/CD36
Wei-Jia Kong	2009	China	Liver cell	InsR, PKC↑	Protein kinase C-dependent upregulation of IR
Qing-Song Xia	2022	China	HFD-fed mice	Ceramide↓	Inhibiting HIF-2α
Hang Zhou	2017	China	Mice macrophage, THP-1 cells	IL-1b, Caspase-1, and mTOR↓	Inhibiting NLRP3 inflammasome activation
			3T3L-1 cells, HFD-fed mice	Activation of AMPK-dependent autophagy	
Zhen-Hua Dong	2021	China	Intralipid-induced murine, HSMCs, AML12 hepatocytes, human umbilical vein endothelial cells	CypD↓	Inhibiting CypD protein expression in skeletal muscle
Lifang Ye	2016	China	HFD-fed mice	TNF-α, IL-6, MCP-1, Ser307↓, and Ser473↑	Inhibiting M1 macrophage activation
Minmin Gong	2021	China	RAW 264.7 and HepG2 cell	p-IRS-1, p-JNK, IL-1B, IL-6, TNF-A, CCL2, and MCP-1↓	Inhibiting the LTB4-BLT1 Axis
Shi-Jun Yue	2018	China	HFD-fed mice	Phosphorylation state of BCKDHA (E1a subunit) and BCKDK	Gut microbiota alteration in BCAA biosynthesis and BCAA catabolism
Ping Yi	2008	China	3T3-L1	IRS-1, PI-3K p85↑	Inhibiting IKKbeta
Lian-Jun Xing	2011	China	NAFLD rat	IRS-2↑	Upregulating IRS-2 mRNA
Yaru Li	2022	China	TNF-α-induced hepatocyte	MEKK1, MEK1/2, and ERK1/2↓	Inhibiting the MEKK1 and MEK1/2 and suppressing their downstream ERK1/2
Tianjiong Lou	2011	China	PA-stimulated HepG2 cell	IL-6, TNF-a↓, and glycogen synthesis↑	Anti-inflammatory activity
Yuanli Wang	2019	China	Dexamethasone-induced 3T3-L1	Glucose usage, adiponectin, and HIF3A↑	Inhibiting HIF3A methylation
Yucheng Li	2020	China	Fructose-fed mice	Leptin↓, PKB/AKT, GSK3β, p-AMPK, and p-LKB1↑	Stimulating the hepatic LKB1/AMPK/PGC1α
Xu Zhang	2012	China	HFD-fed rat	SCFA↑	Structural modulation of the gut microbiota
Marwa El-Zeftawy	2019	Egypt, Saudi Arabia	HFD-fed rat	RBP4↓	Ameliorating PI3K/Akt-p/SIRT-1/PTEN
Ning Zhang	2020	China	Letrozole-induced PCOS	GLUT4↑ and MAPK↓	GLUT4 upregulation *via* PI3K/AKT activation and MAPK suppression
Jia Xu	2022	China	HFD-fed mice and PA-induced hepatocyte	Opa1 ↑	Enhancing mitochondrial architecture *via* the SIRT1/Opa1
Miao Sui	2021	China	PA-stimulated HepG2 cell	NA	Regulating microRNA-146b/SIRT1
Xuhan Liu	2010	China	Type 2 diabetic hamsters	LXRα, PPARα↑, and SREBPs↓	SREBPs, LXRα, and PPARα transcriptional programs
J-J Gu	2012	China	HFD-fed rat	Body weight, visceral fat↓	Preventing alterations IR, IRS-1, and glucagon in β-cells, α-cells, and hepatocytes
Dan Liu	2018	China	HFD-fed rat	TLR4/TNF-α↓, insulin receptor, and insulin receptor substrate-1↑	Modulating gut microbiota along with inhibiting LPS/TLR4/TNF-α
Anyang Li	2022	China	HFD-fed-induced diabetes mellitus	IR, CRP, TNF-a, IKKb, NF-kB, and P65↓	Modulating of IKK/NF-κB, JNK, and IRS-1/AKT in the liver
Ping Yi	2007	China	3T3-L1	NF-kappa B p65↓	Inhibiting nuclear transcription factor-kappa B p65
Guo-Sheng Li	2016	China	HFD-fed hamster	NA	Regulating BMP4 transcriptional
Fen Li	2016	China	HepG2	a7nAChR↑, AChE, pIKKβ Ser181/IKKβ, NF-κB p65, and IL-6↓	Cholinergic anti-inflammatory and inhibiting AChE activity

First, the AMPK activity is activated. BBR appears to reduce IR in H9c2 cardiomyocytes, at least in part, by stimulating AMPK activity, as shown by BBR’s considerably enhanced AMPK activity (Chang et al., 2013). The anti-inflammatory benefits of BBR are achieved by activating AMPK-dependent autophagy in ATMs, hence reducing IR (Zhou et al., 2017). Furthermore, BBR increases the protein expression of phospho-AMP-activated protein kinase (p-AMPK) and protects against IR caused by fructose (Li et al., 2020).

Second, insulin sensitivity and insulin receptor (InsR) are increased. Preliminary findings show that suppressing fat storage and altering the adipokine composition improve insulin sensitivity in human preadipocytes and patients with MetS. BBR increases InsR mRNA and protein expression in human liver cells. Meanwhile, BBR boosts insulin sensitivity, InsR, and protein kinase C (PKC) activity in type 2 diabetes mellitus rat liver (Kong et al., 2009). BBR may reduce IR in rats with non-alcoholic fatty liver disease (NAFLD) by increasing IRS-2 mRNA and protein levels (Xing et al., 2011). In HFD-induced IR rats, BBR may ameliorate the development of IR by differentially preventing alterations of IR, IRS-1, and glucagon in *β*-cells, *α*-cells, and hepatocytes (Gu et al., 2012).

Third, BBR has anti-inflammatory effects. Inflammation also participates in the pathological process of IR. According to a meta-analysis of randomized controlled trials (RCTs) on Chinese subjects with MetS and associated illnesses, high-sensitivity C-reactive protein (hs-CRP) was strongly connected with IR (*r* = 0.9929, *p* < 0.05) (Cao and Su, 2019). BBR efficiently suppressed IL-6 and TNF-α production in palmitate (PA)-stimulated hepatocytes, indicating that BBR may increase insulin sensitivity in conjunction with its anti-inflammatory effects (Lou et al., 2011). BBR inhibited PA-induced NLRP3 inflammasome activation and caspase 1 and interleukin-1 (IL-1) release in HFD-induced IR (Zhou et al., 2017). Additionally, BBR alleviates IR in HepG2 cells *via* a cholinergic anti-inflammatory mechanism (Li F. et al., 2016).

Fourth, the gut microbiota is regulated. BBR improved IR in HFD-fed mice, which was connected not only with gut microbiota changes in branched-chain amino acid (BCAA) production but also with BCAA catabolism in liver and adipose tissues (Yue et al., 2019). The prevention of IR by BBR in HFD-fed rats is at least partially mediated by structural modulation of the gut microbiota, which may help alleviate inflammation by reducing the exogenous antigen load in the host and elevating short-chain fatty acid levels in the intestine (Zhang et al., 2012). BBR reversed dysbacteriosis and inhibited Toll-like receptor 4 (TLR4)/TNF-α activation, resulting in increased InsR and InsR substrate-1 expression in the liver, suggesting that BBR may reduce IR, at least in part by modulating the gut microbiota (Liu et al., 2018).

Fifth, hypoxia-inducible factor (HIF) is inhibited and could alleviate IR by inhibiting fatty acid oxidation (FAO)-mediated activation of the NLRP3 inflammasome (Li X. et al., 2021). In HFD-fed mice, BBR reduced hypoxia-inducible factor 2α (HIF-2α)-induced ceramide production and attenuated ceramide-induced IR (Xia et al., 2022). In IR adipocyte models, BBR improves insulin sensitivity, and the beneficial effects of BBR are possibly realized through the inhibition of HIF3α methylation (Wang et al., 2019).

Finally, other mechanisms are involved, such as reducing PPARG and fatty acid transferase (FAT)/CD36 (Chen et al., 2009), inhibition of cyclophilin protein (Dong et al., 2021), M1 macrophage activation (Ye et al., 2016), the leukotriene B4 (LTB4)–BLT1 axis (Gong et al., 2021), I-kappa-B kinase-β (IKκβ) (Yi et al., 2008), mitogen-activated protein (MAP) kinase kinase kinase (MEKK) 1, MAP kinase (MEK) 1/2, ERK1/2 (Li et al., 2022), nuclear transcription factor-kappa B (NF-κB) p65 (Yi et al., 2007), increased liver X receptor (LXR) α, PPARα, and sterol regulatory element-binding protein (SREBP) (Liu et al., 2010), enhancing mitochondrial architecture *via* sirtuin 1 (SIRT1)/optic atrophy 1 (Opa1) (Xu et al., 2022), regulating microRNA-146b/SIRT1 (Sui et al., 2021), and bone morphogenetic protein 4 (BMP4) transcription (Li G. S. et al., 2016).

### 1.3 BBR and diabetic nephropathy

Hyperglycemia in MetS is composed of either prediabetes or diabetes. The odds ratios (ORs) for isolated microalbuminuria and impaired fasting glucose were 1.58 (1.10–2.25), suggesting that prediabetes, combining impaired fasting glucose and impaired glucose tolerance, may be detrimental to the kidney. Additionally, among individuals with prediabetes, the OR for isolated decreased kidney function was 2.57 (1.31–5.06) (Markus et al., 2018). Without immediate prevention, prediabetes is susceptible to progression into diabetes. Fortunately, BBR slows the progression of prediabetes to diabetes in diabetic fatty rats by enhancing the intestinal secretion of glucagon-like peptide-2 and improving the gut microbiota (Wang et al., 2021).

A meta-analysis of observational studies showed that the overall pooled prevalence of diabetic nephropathy (DN) was 21.8% in China (Zhang X. X. et al., 2020). DN is responsible for 30%–50% of all end-stage renal disease causes. The increased filtration and total renal size of early-stage diabetes are correlated with elevations in glomerular and tubular size. The mesangial compartment of the glomerulus grows as matrix formation rises, likely as the mesangial cell population increases (Ibrahim and Hostetter, 1997).

BBR is frequently used in the prevention and control of DN due to its blood glucose lowering, anti-inflammation, anti-fibrosis, and flora adjustment effects. The comprehensive potential mechanisms of BBR against DN are summarized in Table 3 and are as follows.

**TABLE 3:** BBR and diabetic nephropathy.

Author ID	Year	Country	Model	Molecular	Mechanism
Liping Zhu	2018	China	STZ-induced DN rats, podocyte	IL-1β, IL-6, MCP-1, and apoptosis of podocytes↓	Inhibiting TLR4/NF-κB pathway
Baozhu Ding	2021	China	High-sugar- and high-fat-induced hamsters	IL-1β, IL-6, NLRP3, Caspase-1, GSDMD, Nrf2, and MDA↓	Nrf2-NLRP3-Caspase-1-GSDMD Pathway
Meishuang Zhang	2020	China	HFD-induced DN rats	p-AMPK/AMPK↑, P-ULK/ULK ↓	Inhibiting mesangial matrix expansion and activating autophagy
SiFan Sun	2015	China	HFD and STZ-induced rats	IL-1β, TNF-a, MCP-1, fibronectin, collagen I, and collagen IV↓	Inhibiting NF-κB, TGF-β/Smad3
LiQin Tang	2016	China	High-fat, high-glucose and STZ	ICAM-1, VCAM-1↓, *β*-arrestin 1, *β*-arrestin 2↑	Regulating *β*-arrestin expression and cell adhesion molecule
Zejun Ma	2022	China	HFD and STZ, HK-2 cell	EMT, NLRP3 inflammasome↓	Ameliorating tubulointerstitial fibrosis
Zhong Li	2017	China	STZ-induced	TGF-β, vimentin, and *α*-SMA↓	Inhibiting fibrosis
WeiJian Ni	2022	China	STZ + high glucose-induced mice, GMCs	PI3K-p85, p-Akt, p-AS160, GLUT1, and cyclin D1↓	Regulating abnormal GMC proliferation and the cell cycle
WeiJian Ni	2015	China	STZ	MMP-2↑, MMP-9, TIMP-2, TGF-β, fibronectin, and collagen IV↓	Regulating the MMP/TIMP system
Guannan Yang	2017	China	KKAy mice, renal tubular epithelial cells	E-Cadherin, *α*-SMA↑, EMT, jagged1, notch1, hes1, and snail1↓	Inhibiting EMT through Notch/snail pathway
Kaipeng Huang	2012	China	GMCs, STZ-induced rats	S1P2 and FN↓	Suppressing the S1P–S1P2 receptor
Sheng Liu	2012	China	STZ-induced rats	TGF-β, Smad2/3↓, SnoN, and Smad7↑	Maintaining the dynamic balance in TGF-beta1/SnoN

First, the NF-κB pathway is inhibited. BBR decreased renal impairment in the streptozotocin (STZ)-induced DN rat model, as shown by lowering fasting blood glucose, kidney weight to body weight ratio, 24-h proteinuria, urea nitrogen (BUN), and creatinine (Cr) levels. BBR suppressed the TLR4/NF-κB pathway and decreased the systemic and renal cortex inflammatory responses in STZ-induced DN rats and high glucose (HG)-induced podocytes (Zhu L. et al., 2018). Another study revealed that under diabetic conditions, BBR lowers fibronectin (FN) expression by acting on the sphingosine 1-phosphate (S1P) 2 receptor in the mesangium, which might be related to its inhibitory action on NF-κB activation (Huang et al., 2012). A current study shows that BBR can prevent type 2 diabetes by suppressing NF-κB-driven renal inflammation and the transforming growth factor (TGF)/Smad3 signaling pathway (Sun et al., 2015).

Second, mesangial proliferation was reduced. Glomerular mesangial cell proliferation is one of the main pathological changes in DN. A current study found that Huang-Gui solid dispersion (HGSD), a novel BBR formulation, prevented DN by reducing renal mesangial matrix growth and activating autophagy, which might be linked to AMPK phosphorylation activation (Zhang M. et al., 2020). Furthermore, BBR can prevent the progression of DN, perhaps by blocking the PI3K/Akt/AS160/glucose transporter 1 (GLUT1) signaling pathway, which regulates HG-induced aberrant glomerular mesangial cell (GMC) proliferation and the cell cycle (Ni et al., 2022).

Third, BBR has anti-inflammatory effects. In the DN hamster kidney, NLRP3–Caspase-1–Gasdermin D (GSDMD) signaling was increased. BBR can diminish oxidative stress damage by modulating antioxidative Nrf2 and then NLRP3–Caspase-1–GSDMD signaling to prevent pyroptosis and antagonize DN inflammation-induced damage (Ding et al., 2021). BBR inhibited HG-induced epithelial-to-mesenchymal transition (EMT) and renal interstitial fibrosis by inhibiting the NLRP3 inflammasome, implying that BBR might be utilized as a new medication to treat tubulointerstitial fibrosis in DN (Ma et al., 2022).

Fourth, BBR has anti-fibrotic effects. Glomerular mesangial cell proliferation is one of the main pathological changes in DN. Consistently, BBR inhibits renal tubular EMT and renal interstitial fibrosis through Notch/snail pathway regulation (Yang et al., 2017). Through the Smad signaling pathway, BBR can maintain the dynamic equilibrium of TGF-β1/SnoN expression in renal tissues, hence mitigating renal dysfunction (Liu et al., 2012).

Finally, there are other mechanisms. The renoprotective effects of BBR on DN may be connected with alterations in the extracellular matrix *via* control of the matrix metalloproteinase (MMP)/tissue inhibitor of metalloproteinase (TIMP) pathway in the rat kidney (Ni et al., 2015). BBR (100, 200 mg/kg) alleviated DN histopathology, which may be related to the control of *β*-arrestin expression, as well as intercellular cell adhesion molecule-1 (ICAM-1) and vascular cell adhesion molecule-1 (VCAM-1) levels in the rat kidney (Tang et al., 2016).

### 1.4 BBR and hypertension nephropathy

In MetS, high blood pressure is defined as prehypertension or hypertension according to varying diagnosis criteria. A meta-analysis revealed that prehypertension showed an increased risk of chronic kidney disease (CKD) (pooled RR = 1.28) compared with the optimal BP values. Therefore, prehypertension is a potential cause of CKD (Li Y. et al., 2016). Meanwhile, hypertension and malignant hypertension can induce hypertensive kidney damage with different kidney pathological features. Secondary hypertension is commonly induced by parenchymal and renovascular diseases. Thus, hypertension and renal injury interact, ultimately cultivating a vicious cycle. Anti-hypertension is especially crucial for blood pressure-dependent kidney damage, regardless of which reason induces hypertension.

Abnormal hemodynamics, activation of the renin–angiotensin–aldosterone system (RAAS), oxidative stress, inflammation, genetic factors, and metabolic disorders contribute to hypertensive kidney damage (Fang-Ming et al., 2015). The pathological features of benign hypertension nephropathy are characterized by a thickened interlobular artery intima, stratified elastic layer, and arteriole hyalatosis, with/without media smooth muscle cell proliferation. Pathological manifestations of malignant hypertension kidney injury are malignant lesions of the arteries, including fibrinous necrosis of the arteries (acute lesions), “scallion-skin-like” changes in intimal hyperplasia of the arteries (chronic lesions), fibrinous necrosis of glomerular vascular loops, and benign lesions of the arteries (Shao-Shan et al., 2015).

BBR exerts certain beneficial effects on hypertension nephropathy. A randomized, double-blind, placebo-controlled clinical trial indicated a significant decrease in systolic blood pressure (SBP) (123 ± 7 *vs*. 115 ± 9 mmHg, *p* < 0.01), which was observed after BBR administration compared with placebo (Perez-Rubio et al., 2013). The anti-hypertension mechanisms of BBR are summarized in Table 4 and are as follows.

**TABLE 4:** BBR and hypertension nephropathy.

Author ID	Year	Country	Model	Targeted molecular	Mechanism
Limei Liu	2015	China	Spontaneously hypertensive rats	eIF2a, ATF3, ATF6, XBP1, COX-2, and ROS↓	Activating AMPK, thus inhibiting ER stress and scavenging ROS
Zhichao Wang	2022	China	Angiotensin II-induced hypertensive mice	FMO3 and TMA/TMAO↓	Regulating the gut microbiota
Yu-Guang Ma	2017	China	STZ-induced rats	BKCa β1-subunit↑	Activating BKCa channel improved vasodilation
Gaoxing Zhang	2019	China	Spontaneously hypertensive rats	EMPs, aPWV↓, EPC numbers, and CFUs↑	Reduce endothelial injury and arterial stiffness
Hua Tian	2019	China	One-clip (2K1C) renovascular hypertensive rats	MAP, PVN Fra-like activity, NE, reduced NOX2, NOX4, Erk1/2, iNOS↓	ROS/Erk1/2/iNOS

First, endothelial function is improved. Endothelial dysfunction is an important determinant risk factor for the development of hypertension and its complications. Hence, improving endothelial function has major clinical importance. In spontaneously hypertensive rats (SHRs) with carotid arteries, BBR lowers endothelium-dependent contractions (EDCs), most likely by activating AMPK, which then prevents endoplasmic reticulum stress and scavenges reactive oxygen species (ROS), causing cyclooxygenase-2 (COX-2) to be downregulated (Liu et al., 2015). Endothelial dysfunction and arterial stiffness are linked to abnormal alterations in circulating microparticles (MPs) and endothelial progenitor cells (EPCs) in SHRs. BBR enhanced endothelial function by preserving superior endothelium-dependent vasodilation and retained arterial elasticity by reducing aortic pulse wave velocity (aPWV) and increasing the content of arterial media elastin fiber (Zhang G. et al., 2020).

Second, there is vasodilation. By activating the large conductance calcium-activated K+ 504 channels (BKCa) in vascular smooth muscle cells (VSMCs), BBR decreased blood pressure and enhanced vasodilation in diabetic rats, suggesting that BBR could provide a combined treatment for regulating hyperglycemia and blood pressure in diabetes (Ma et al., 2017). The angiotensin-converting enzyme inhibitory impact, direct release of nitric oxide/cyclic guanosine monophosphate (NO/cGMP), and α1-adrenoreceptor antagonistic activity of BBR all contribute to its vasodilatory effect, which may explain the drop in blood pressure (Derosa et al., 2012).

Third, BBR affects antioxidants. In 2K1C renovascular hypertensive rats, chronic infusion of BBR decreased mean arterial pressure (MAP), paraventricular nucleus Fra-like activity, and plasma levels of norepinephrine (NE), as well as NADPH oxidase 2 (NOX2), NOX4, ERK1/2, and inducible nitric oxide synthase (iNOS). This finding suggests that BBR attenuates hypertension and sympathoexcitation by inhibiting the ROS/ERK1/2/iNOS pathway (Tian et al., 2019).

Finally, the gut microbiota is regulated. BBR administration dramatically reduced vascular dysfunction and pathological remodeling in Ang II-induced hypertensive mice, suggesting that the protective effect of BBR in hypertension may be attributable (at least in part) to the reduction in trimethylamine (TMA)/trimethylamine-N-oxide (TMAO) formation through gut microbiota regulation (Wang Z. et al., 2022).

However, a systematic review involving 614 participants revealed that the data from randomized trials are insufficient to prove the efficacy and safety of BBR in the treatment of hypertension as its evidence is restricted, of low quality, and ultimately inconclusive (Suadoni and Atherton, 2021).

### 1.5 BBR and hyperlipidemia

Hyperlipidemia is frequently secondary to nephrotic syndrome, chronic renal failure, and postrenal transplant conditions. Because these disorders appear to enhance the risk of coronary heart disease, lowering blood lipids is particularly important (Grundy, 1990). Moreover, feeding cholesterol to experimental animals induced the initiation and progression of glomerular injury, and treatment of hyperlipidemic animals with lipid-lowering drugs slowed the development of glomerulosclerosis (Kamanna et al., 1998).

BBR, a natural lipid-lowering agent, is salutary in animal and clinical experiments. Scholars showed that BBR improved adipose tissue remodeling by activating Sirtuin 3 (SIRT3), which could contribute to the anti-hyperlipidemic effect (Li 2022). After 8 weeks of treatment with BBR, cholesterol was significantly decreased in a rat model of MetS (Li et al., 2015). Patients who received BBR also showed statistically significant improvements in total cholesterol (MD, −0.58; 95% CI, −0.74 to −0.41) and low-density lipoprotein (LDL) (MD, 0.52; 95% CI, −0.68 to −0.35) in antipsychotic-associated weight gain and MetS in patients with schizophrenia (Chan et al., 2022). In hyperlipidemic patients with chronic hepatitis or liver cirrhosis, BBR significantly lowered blood cholesterol, triglycerides, and LDL-cholesterol (LDL-c) (Zhao et al., 2008). The exhaustive potential hypolipidemic mechanisms of BBR are shown in Table 5 and are as follows.

**TABLE 5:** BBR and hyperlipidemia.

Article ID	Year	Country	Model	Targeted molecular	Pathway
Jean-Marie Brusq	2006	France	HepG2 human hepatoma cells	AMPK phosphorylation and AMPK activity↑	Increasing AMPK activity
PING CHEN	2021	China	Non-alcoholic fatty liver disease (NAFLD) rats	TG, ALT, AST, TC, TG, LDL↓, and MTTP↑	Reversing the abnormal expression of MTTP and LDLR
You-Jin Choi	2017	Korea	HepG2 human hepatoma cell, mouse hepatocytes	CD36 transcription↑	Activating AMPK induces transcriptional activation of CD36
Jia-Ge Dai	2021	China	Porcine oocytes	FABP3, SREBF1, PPARG↓, PPARG phosphorylation↑, and JNK phosphorylation↓	Activating miR-192
JiaGe Dai	2021	China	Porcine oocytes	miR-192↑, SREBF1, and PPARG↓	Activating miR-192
Xinyi Fang	2022	China	Mice with disturbances in glucose and lipid metabolism	NA	Changing gut microbiota and metabolites
Shenghua Gu	2015	China	A high-fat-diet-induced hamster hyperlipidemia model	Bile acids↑, CYP7A1 expression	The turnover and enterohepatic circulation of bile acids and intestinal farnesoid X receptor signal pathway
Woo Sik Kim	2009	Korea	Obese mice	AMPK↑	Increasing AMPK activity
Hui Liang	2018	China	QSG-7701 hepatocytes and mice	ABCA1↑	Increasing ABCA1 protein levels through PKCδ to reduce the phosphorylation of serine residues in ABCA1
Livia Pisciotta	2012	Italy	Familial Hypercholesterolemia heterozygotes	PCSK9, LDLRs↓	Increasing expression and stability of LDLRs and/or suppressing PCSK9 expression
Gang Ren	2020	China	HepG2 cells	AMPKa1	AMPKa1
Qingfeng Rong	2022	China	Type 2 diabetic db/db mice	CPT1, ACOX1, PPAR-α↑	Improving high-glucose-induced reduction of fatty acid oxidation
Runbin Sun	2017	China	Intestine-specific FXR knockout (FXRint2/2) mice	BSH↓, TCA↑,FXR↑, CD36↓	Intestinal FXR signaling pathway
Can Wang	2016	China	HepG2 cells	AMPK↑	Increasing AMPK activity
Shengnan Wei	2016	China	Type 2 diabetic (T2D) mice, HepG2 cells	HNF-4α↓ and miR122↓	Decreasing expression of HNF-4α and miR122
Sa Yang	2022	China	HFD-fed mice and oleic acid-treated HepG2 cells	ATGL, GK, PPARα, CPT-1, ACC1, FAS, and CD36	Regulating the protein expression of ATGL, GK, PPARα, CPT-1, ACC1, FAS, and CD36
Shuangshuang Yao	2020	China	Db/db male mice	PGC-1α↑	AMPK/PGC-1α
Muyu Yu	2021	China	Male C57BL/6J mice	ComplexⅠ↓	Repressing complexⅠ
Qian Zhang	2011	China	KKAy mice	GLUT4, MAPK14, MAPK8, PPARα, UCP2, HNF4α↑, PPARγ, CCAAT/CEBP, PGC 1α, and resistin↓	AMPK p38 MAPK-GLUT4, JNK, and PPARα
Yan Zhou	2014	China	Human hepatoma HepG2 cells	LDLR↑	Upregulating LDLR expression
Xiaofei Zhu	2018	China	HepG2 cells	LDLR↑	Upregulating LDLR expression

First, BBR inhibits lipid synthesis in human hepatocytes through the direct and indirect activation of AMPK in peripheral tissues (notably the liver and muscles) (Brusq et al., 2006; Kim et al., 2009; Wang et al., 2016). BBR can protect the lean body mass from excessive lipid accumulation by promoting mitochondrial biogenesis and improving FAO in an AMPK/PGC-1α-dependent manner (Yao et al., 2020). BBR moderates lipid metabolism through AMPk-p38 mitogen-activated protein kinase (MAPK)-GLUT4 (Zhang et al., 2011). In addition, AMPKα1 is essential for BBR to improve glucose and lipid metabolism in HepG2 cells (Ren et al., 2020). However, prolonged activation of AMPK increases CD36 expression in hepatocytes, resulting in fatty acid uptake linked to hepatocellular lipid accumulation and fatty liver (Choi et al., 2017).

Second, BBR and its metabolites exhibit lipid-lowering effects by upregulating LDL receptor (LDLR) expression (Pisciotta et al., 2012; Zhou et al., 2014; Zhu X. et al., 2018). A study demonstrated that fatty liver could be improved by BBR administration by reversing the abnormal expression of microsomal triglyceride transfer protein (MTTP) and LDLR and inhibiting lipid synthesis (Chen et al., 2021).

Third, BBR could significantly modify the structure and composition of gut microbiota (Yang et al., 2022), and the changes in gut microbiota and metabolites are correlated with BBR improving lipid metabolism disturbances (Fang et al., 2022). Other studies demonstrated that BBR significantly inhibited the 7α-dehydroxylation conversion of cholic acid to deoxycholic acid, and the hypocholesterolemic effect of orally administered BBR was involved in its effect on modulating the turnover of bile acids and the farnesoid X receptor signaling pathway (Gu et al., 2015; Sun et al., 2017).

Fourth, BBR exhibits a dual effect on maintaining lipid homeostasis through hepatocyte nuclear factor (HNF)-4α-regulated miR-122 expression (Wei et al., 2016). BBR promotes lipid metabolism by activating the expression of miR-192, downregulating steroid regulatory element binding transcription factor 1 (SREBF1) and PPARG, increasing PPARG phosphorylation, and reducing JNK phosphorylation (Dai J. et al., 2021a; Dai J. G. et al., 2021b).

Finally, BBR alleviates lipid deposition by improving the HG-induced reduction in FAO (Rong et al., 2021). BBR can reduce steatosis by increasing ATP-binding cassette transporter A1 (ABCA1) protein levels through PKCδ to reduce the phosphorylation of serine residues in ABCA1 (Liang and Wang, 2018). Moreover, BBR represses complex I in the gut and liver and consequently inhibits lipid metabolism, leading to alleviation of obesity and fatty liver (Yu et al., 2021).

### 1.6 BBR and hyperuricemia-related kidney disease

Hyperuricemia does not belong to the scope of MetS. However, hyperuricemia is induced by abnormal metabolism and is closely linked to MetS. Hence, hyperuricemia-related kidney disease is discussed under this condition. The mechanisms of hyperuricemia-related kidney disease involve RAAS activation, direct damage to uric acid (UA) crystals, and inflammation. The pathological feature of hyperuricemia-related kidney disease is characterized by tubule-interstitial injury. BBR exerts antihyperuricemic and nephroprotective effects in hyperuricemic kidney disease (Li X. et al., 2021). The mechanisms of BBR in hyperuricemia and related kidney disease are listed below.

First, BBR lowers UA by inhibiting UA biosynthesis and promoting UA excretion. BBR suppresses the expression of xanthine oxidase (XOD), urate transporter 1 (URAT1), and glucose transporter 9 (GLUT9) to lower UA (Shan et al., 2022). In addition, BBR effectively reduced serum UA levels in hyperuricemic rats by increasing urine uric acid levels and urate fractional excretion.

Second, BBR has anti-inflammatory effects. Inflammation is involved in hyperuricemia-related kidney injury. BBR drastically reduced the levels of UA, BUN, and Cr in a mouse model of hyperuricemia created by potassium oxonate and hypoxanthine, as well as kidney injury. BBR inhibits the activation of the NLRP3 inflammasome and decreases TNF-α (Shan et al., 2022), IL-1β (Liu et al., 2016), and IL-18 levels, as well as NLRP3, ASC, caspase-1, and URAT1 expression (Li Q. et al., 2021).

Finally, the gut microbiota is regulated. Subsequently, 16S rRNA sequencing data showed that BBR enriched the abundance of *Coprococcus*, *Bacteroides*, *Akkermansia*, and *Prevotella* in potassium oxonate-induced hyperuricemia, clarifying that BBR ameliorates hyperuricemia by modulating the gut microbiota (Shan et al., 2022).

## 2 Discussion

MetS-associated kidney diseases can be induced by different components of MetS, such as obesity-related ORG, hypertension-related hypertension nephropathy, hyperglycemia-related DN, hyperlipidemia and hyperlipidemia-associated kidney injury, and hyperuricemia and hyperuricemia-associated kidney disease. One or several components of MetS can contribute to kidney damage alone or together with other elements. Hence, the clinical manifestation and pathological features of MetS-associated kidney disease are different according to the component(s) involved. In addition, the pathological features are not always consistent with clinical manifestations. Therefore, the specific component may not cause this component-associated kidney disease. In this situation, renal biopsy is a unique way to diagnose MetS-associated kidney disease. Due to the complexity of MetS, its therapy is renal pathology-oriented.

Regarding renal pathology, MetS can lead to glomerulopathy (associated with obesity and disorders of glucose and lipid metabolism), lesions of the small arteries of the kidney (associated with hypertension and diabetes), and tubule-interstitial lesions (associated with hyperuricemia and secondary to renal arterioles and glomerulus lesions) (Yi-Pu, 2006). Thus, renal biopsy is crucial for the precise diagnosis of MetS-associated kidney diseases. The treatment plan varies depending on different kidney lesions.

**FIGURE 5:**
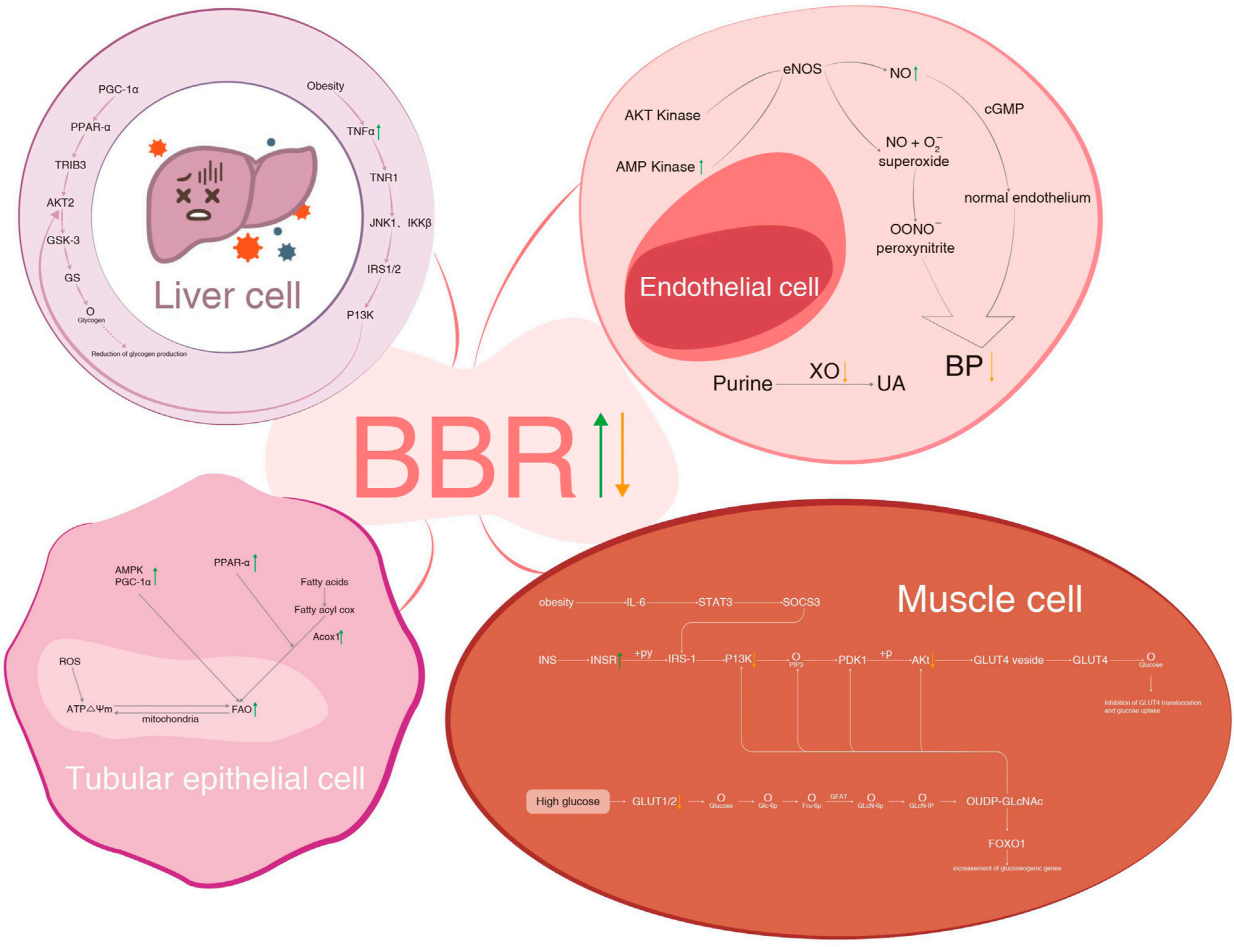
BBR on metabolic pathways of IR in liver cell and muscle cells, hypertension and hyperuricemia in endothelial cells, and hyperlipidemia in tubular epithelial cells. BBR, berberine; IR, insulin resistance; PGC-1α, peroxisome proliferator-activated receptor-γ coactivator 1α; PPAR-α, peroxisome proliferator-activated receptor α; GLUT4, glucose transporter 4; TR1B3, tribbles homologue 3; AKT2, RAC serine/threonine-protein kinase; GSK-3, glycogen synthase kinase-3; GS, glycogen synthase; TNF-α, tumor necrosis factor-α; TNR1, tumor necrosis factor receptor 1; JNK1, c-Jun-N-terminal kinase 1; IKKβ, inhibitor of nuclear factor kappa-B kinase subunit beta; IRS1/2, insulin receptor substrate 1/2; P13K, phosphatidylinositol-3-kinase; AMP, adenosine 5'-monophosphate activated protein; cGMP, cyclic guanosine monophosphate; ROS, reactive oxygen species; Acox1, acyl-coenzyme a oxidase 1; IL-6, interleukin-6; STAT3, signal transducer and activator of transcription 3; SOCS3, suppressor of cytokine signaling 3; INS, insulin; INSR, insulin receptor; +py, tyrosine phosphorylation; PDK1, phosphoinositide-dependent kinase-1; GLUT4 vesicle, glucose transporter 4 vesicle; GFAT, glutamine fructose phosphate transaminase; FOXO1, forkhead box O1.

In this review, we summarized the effects of BBR on different components of MetS and its kidney injury. More attention was given to improving IR, lipid-lowering, and glucose-lowering than to controlling obesity, hypertension, and hyperuricemia. On the one hand, the levels of insulin, lipids, and glucose are easy to determine and show efficacy in the short term. On the other hand, the effects of losing weight and lowering blood pressure are difficult to realize unless IR, lipids, and glucose are well-controlled. The antihyperuricemic effect of BBR is rarely reported, possibly because it is beyond the scope of MetS. Anti-inflammation and modulation of gut microbiota are common mechanisms of BBR, which participate in intervening in nearly all components of MetS. The diagram of BBR on metabolic pathways of IR in liver and muscle cells, hypertension and hyperuricemia in endothelial cells, and hyperlipidemia in the tubular epithelial cell is shown in Figure 5.

Meanwhile, several limitations and recommendations for these included studies should be noted. First, different modeling methods are applied in different components; even for the same component, varying modeling methods are used. In addition, the same modeling method is employed in different components. For example, obesity, diabetes, and IR models could be established by HFD. Therefore, the efficacy and severity of different modeling methods are difficult to estimate, as are the efficacy and safety of BBR. Second, few studies on MetS-associated kidney disease have been reported due to a lack of awareness among clinicians. We found that MetS-associated kidney disease is replaced by isolated component-induced kidney damage, such as DN, ORG, and hypertension nephropathy. Third, renal biopsy is not performed in most studies. Although clinical renal lesions are presented, including proteinuria and abnormal BUN or Cr, the gold standard for kidney injury tends to be ignored. Based on the renal biopsy, the final pathological diagnosis of diabetic patients is possibly non-diabetic nephropathy. If these diabetic patients are treated according to DN, kidney diseases may be delayed or aggravated. Fourth, altered purity, doses, duration, and administration routes of BBR dependent on different design protocols are responsible for the variance of effects. Fifth, the bioavailability of BBR is rather low after it is absorbed by the gastrointestinal tract. After a single oral dose of 400 mg of BBR, the highest concentration (Cmax) of BBR in human plasma is 0.4 ng/ml (Hua et al., 2007). BBR is commonly applied at high doses to increase Cmax, which may induce adverse gastrointestinal effects. Consequently, novel formulations and derivatives or analogs of BBR may enhance bioavailability. Sixth, most recent studies concerning BBR are derived from cells or animals, and the definitive efficacy and safety of BBR urgently need to be further confirmed in large-scale, high-quality, multicenter RCTs. Finally, most of the research is from China, so race bias may be an issue.

BBR is believed to be omnipotent for different organs and tissues’ illnesses, which makes us concerned if its efficacy is exaggerated to some extent. However, BBR properties are based on its beneficial effects on dysmetabolism, so it is acceptable in some way. Nonetheless, it may be long before BBR is available for patients with dysmetabolic diseases, including MetS and related kidney injuries.

## 3 Conclusion

Overall, this review provides an important account of the impact of BBR on MetS and its nephroprotective effects. The “one-drug with multiple mechanisms and multiple targets” property of BBR enables its applications in obesity, IR, hypertension, hyperglycemia, hyperlipidemia, and hyperuricemia. Among these abundant mechanisms of BBR, anti-inflammation and regulation of gut microbiota seem to be the common mechanisms. Due to its low cost, easy obtainability, efficacy, and safety, BBR is promising in MetS-associated kidney disease, especially in developing countries.
